# Highly enhanced response of MoS_2_/porous silicon nanowire heterojunctions to NO_2_ at room temperature†

**DOI:** 10.1039/c7ra13484c

**Published:** 2018-03-21

**Authors:** Shufen Zhao, Zhengcao Li, Guojing Wang, Jiecui Liao, Shasha Lv, Zhenan Zhu

**Affiliations:** State Key Laboratory of New Ceramics and Fine Processing, School of Materials Science and Engineering, Tsinghua University Beijing 100084 China zcli@mail.tsinghua.edu.cn; Key Laboratory of Advanced Materials (MOE), School of Materials Science and Engineering, Tsinghua University Beijing 100084 China; Department of Engineering Physics, Tsinghua University Beijing 100084 China

## Abstract

Molybdenum disulfide/porous silicon nanowire (MoS_2_/PSiNW) heterojunctions with different thicknesses as highly-responsive NO_2_ gas sensors were obtained in the present study. Porous silicon nanowires were fabricated using metal-assisted chemical etching, and seeded with different thicknesses. After that, MoS_2_ nanosheets were synthesized by sulfurization of direct-current (DC)-magnetic-sputtering Mo films on PSiNWs. Compared with the as-prepared PSiNWs and MoS_2_, the MoS_2_/PSiNW heterojunctions exhibited superior gas sensing properties with a low detection concentration of 1 ppm and a high response enhancement factor of ∼2.3 at room temperature. The enhancement of the gas sensitivity was attributed to the layered nanostructure, which induces more active sites for the absorption of NO_2_, and modulation of the depletion layer width at the interface. Further, the effects of the deposition temperature in the chemical vapor deposition (CVD) process on the gas sensing properties were also discussed, and might be connected to the nucleation and growth of MoS_2_ nanosheets. Our results indicate that MoS_2_/PSiNW heterojunctions might be a good candidate for constructing high-performance NO_2_ sensors for various applications.

## Abstract 2

Molybdenum disulfide/porous silicon nanowire (MoS_2_/PSiNW) heterojunctions with different thicknesses as highly-responsive NO_2_ gas sensors were obtained in the present study.

## Graphical abstract

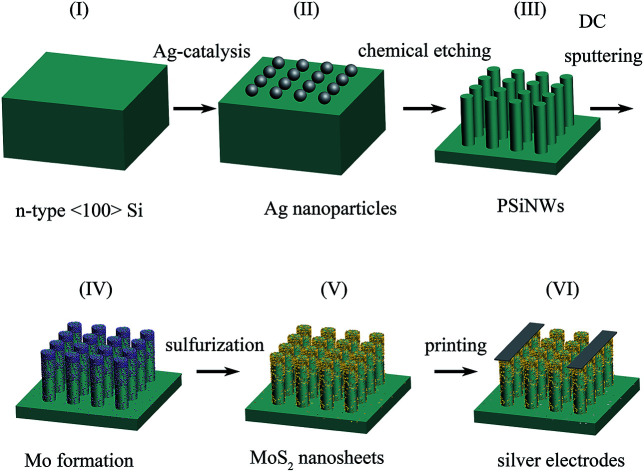

## 1. Introduction

Gas sensors play an important role in monitoring the environment and detecting air pollution. Many factors affect the gas sensing capacity of a gas sensor, including the materials, sensing mechanism and environmental effects (temperature, humidity).^1,2^ Recently, many researchers have devoted their efforts to developing sensors with high sensitivity and low operating temperatures.^3^ It is well known that semiconducting materials are used in gas sensors, such as metal oxides, silicon, and two-dimensional (2D) materials. Metal oxides are a common material to be employed for gas detection.^4–6^ However, they exhibit poor conductivity and require high temperatures to operate.^7^ In particular, silicon and two-dimensional (2D) materials have been widely used in low-temperature sensors.^8,9^

Silicon has many advantages including stability, abundance and optical properties, and it can be employed in microelectronic technologies.^10^ Among these silicon-based materials, PSiNW materials are one of the most promising n-type semiconductors, as they possess large surface areas and high chemical reactivity.^11^ For these reasons, they are widely used in gas sensing devices. PSiNWs are impressive as they can be easily integrated with other semiconductors to lower the operating temperature of the resulting gas sensor.^12^ However, there are several disadvantages, such as poor sensitivity and oxidation of the surface of the material at room temperature.

2D materials such as graphene and MoS_2_ are developing rapidly, with high surface-to-volume ratios.^13^ MoS_2_ has a 2D layered structure and its band gap is affected by the number of stacked layers.^14^ Monolayer MoS_2_ is an n-type semiconductor with a direct band gap of ∼1.9 eV, while bilayer and thicker MoS_2_ crystals have indirect band gaps of ∼1.3 eV.^15,16^ MoS_2_ is used as a gas sensor mainly because of its various sites (sulfur defects, vacancies, and edge sites).^17^ Thermal treatments can improve device performance.^18^ Moreover, compared with other synthetic methods, CVD has the obvious advantages of thickness control^19,20^ and the ability for large-scale growth of MoS_2_.^21^

MoS_2_ could act as a protecting layer for PSiNWs against oxidation damage when applied to gas sensor devices. However, few studies have reported application of MoS_2_/PSiNW heterojunctions to gas sensing. In this paper, MoS_2_/PSiNW heterojunctions fabricated on a substrate with Ag electrodes are presented. The effects of the thickness and deposition temperatures on the gas sensing properties were also explored. As a consequence, MoS_2_/PSiNW heterojunctions with different morphologies showed high sensitivity, low operating temperatures and fast response/recovery properties. This study represents an important step to improve gas sensing properties through the synthesis of MoS_2_/PSiNW heterojunctions.

## 2. Experimental section

### 2.1. Sensor fabrication process

**Fig. 1:**
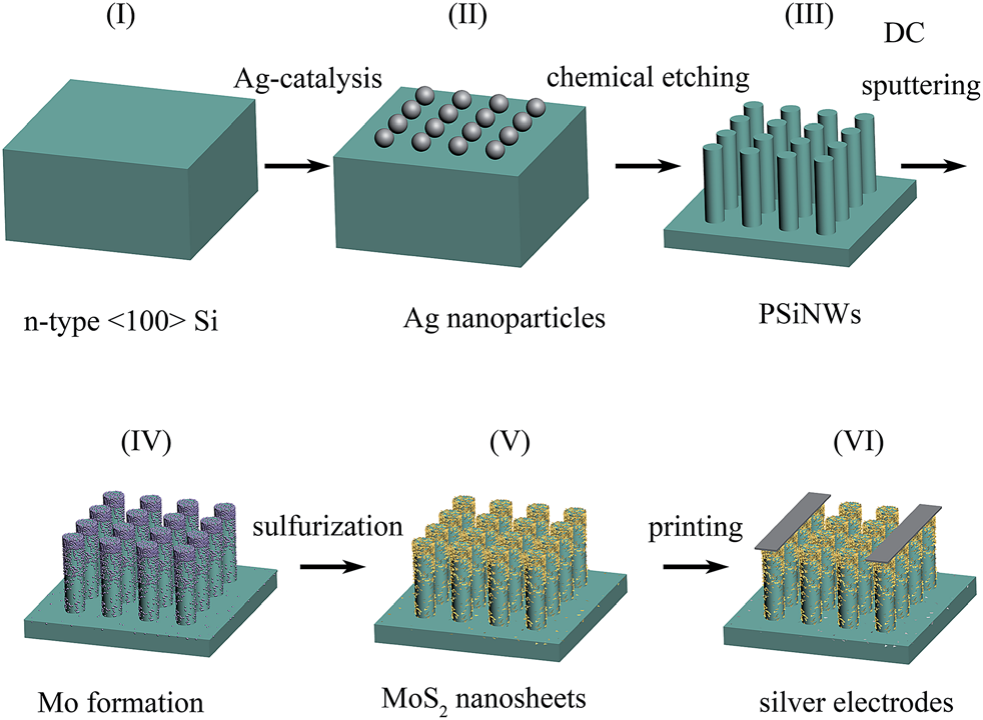
Fabrication process of the MoS_2_/PSiNW-based sensor devices.

MoS_2_/PSiNW heterojunctions were fabricated on 〈100〉 orientation, n-type Si substrates using Ag-assisted chemical etching, direct current (DC) magnetic sputtering and chemical vapor deposition (CVD) methods, as shown in Fig. 1. The fabrication process is further explained as follows. The silicon wafers were cut and ultrasonically cleaned in acetone, ethanol and deionized water for 15 min each. Then they were immersed in boiling H_2_SO_4_/H_2_O_2_ solution at 135 °C for 1 h to remove the organic contaminants. To remove the silicon oxide layer, they were treated with 5% HF before use. PSiNWs were synthesized using a metal-assisted chemical etching method. Pre-treated silicon wafers were placed into a mixed solution of 4.8 M HF and 5 mM AgNO_3_ to obtain a layer of Ag nanoparticles, and were then immersed into a solution of 4.8 M HF and 0.3 M H_2_O_2_ at 30 °C. Ag nanoparticles on the silicon substrate play an important role as catalysts in the etching process. An etching time of 15 min was selected, and we obtained PSiNWs after the etching process was finished. To remove residual Ag nanoparticles and the silicon oxide layer, the PSiNWs were dipped in HNO_3_ solution and HF solution successively. After each procedure, they were washed with plenty of water.

Mo atoms were deposited onto the PSiNW substrate using direct current (DC) magnetic sputtering, which could control different thicknesses. The deposition was carried out with 70 W power and a working Ar pressure of 0.3 Pa, and the base pressure was below 10^−4^ Pa. We selected three parameters of the magnetic sputtering time for the Mo seed layers (1 min, 3 min and 5 min), used to control the thickness of the Mo seed layer on the PSiNW substrate.

MoS_2_ nanosheets were grown on PSiNWs using chemical vapor deposition (CVD). After the deposition of seed layers on the PSiNW substrates, the substrates were placed at the center of the furnace and sulfur powder (1.5 g) was placed at the upstream end. Two temperature ranges were used to separate the PSiNW substrates covered by Mo seed layers and sulfur powder. Sulfur powder (1.5 g) was placed upstream of the quartz tube at a distance of 10 cm from annealing furnace. At the beginning, a PSiNW substrate covered by Mo seed layers was put on a quartz boat, which was still downstream of quartz and outside of the annealing furnace. The annealing furnace needed to be checked for air tightness and N_2_ was injected to get rid of residual air. The temperature of the center zone was increased to 770 °C at a ramp rate of 14 °C for the growth process and the upstream zone was heated to 130 °C at a ramp rate of 26 °C for vaporization of elemental sulfur powders. After the center zone had risen to 770 °C and the upstream zone had been maintained at 130 °C, the quartz boat was pushed into the center of the furnace with a magnet and maintained for 90 min. Finally, the samples were moved out of the furnace after natural cooling. During whole process, the flow rate of Ar carrier gas was maintained at 200 standard cubic centimeters per minute (SCCM). We marked samples as “MoS_2_/PSiNWs-1 min”, “MoS_2_/PSiNWs-3 min” and “MoS_2_/PSiNWs-5 min”, according to the magnetic sputtering time (1 min, 3 min and 5 min, respectively) of the Mo seed layers. To study the effect of deposition temperature in the chemical vapor deposition process on the gas sensing behaviors, three distinct MoS_2_/PSiNW heterojunctions with different deposition temperatures of 720 °C, 770 °C and 820 °C were synthesized, named “S-1”, “S-2” and “S-3”, respectively.

### 2.2. Characterization

The surface morphologies and microstructures were characterized using scanning electron microscopy (SEM, JEOL-JSM 7001F, Tokyo, Japan) and high-resolution transmission electron microscopy (HRTEM, JEM-2100F). X-ray photoelectron spectroscopy (XPS, ESCALAB250Xi, Thermo, Waltham, MA, America) used the C 1s signal (284.8 eV) as a reference to calibrate the binding energy. X-ray diffraction (XRD) patterns were obtained on a Rigaku Smart Lab (Tokyo, Japan) and Raman spectra were obtained using a Lab Ram HR Evolution Micro Raman Spectrometer. These patterns and spectra were used to analyze the structural features and phase purity of the MoS_2_/PSiNW heterojunctions. Raman microscopy was performed with an excitation wavelength of 532 nm.

### 2.3. Gas sensing measurements

To measure the gas sensing properties of the samples, two silver electrodes were deposited onto them using printing, and connected to a digital resistance measurement system. Then the experiment was carried out in a home-made system. The testing chamber used was a 250 mL metal chamber, and gas would fill the chamber in 30 seconds based on the flow rate used. Gases used for the test were NO_2_ (0.01% NO_2_ with 99.99% N_2_) and pure N_2_ (99.999%). At the beginning, pure N_2_ was needed to inject into the chamber. After the resistance of the samples reached a stable value, gases were mixed to obtain certain concentrations and injected into the chamber until a new constant value of the concentration was achieved. The measuring system recorded the resistance of the sensors at all times. By controlling the mass flow to change the specific gas concentration, the computer recorded the resistance change of the samples *versus* the concentration of NO_2_. The gas filled the chamber within few seconds and such a setup was found to be appropriate for the gas sensing measurements. Response was one of main gas sensing properties, which is defined as follows:
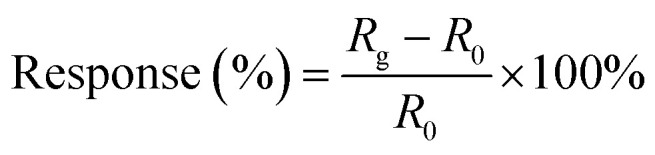
where *R*_g_ is the measured electric resistance of a gas sensor at a certain concentration of NO_2_ and *R*_0_ is the resistance in a pure N_2_ atmosphere.

## 3. Results and discussion

**Fig. 2:**
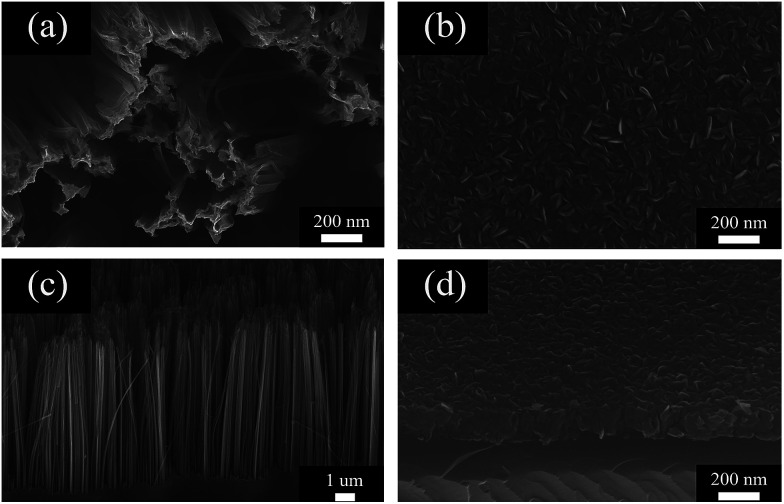
SEM images of PSiNWs: (a) top view (c) side view. SEM images of MoS_2_ nanosheets on wafers: (b) top view (d) side view.

Scanning electron microscopy (SEM) was used to characterize the morphology of the samples. Fig. 2a and c show the top view and side view of the PSiNWs obtained *via* Ag-assisted chemical etching. The surface morphology of the PSiNWs is rough and the tip parts are clustered. The obtained porous silicon nanowires are arranged vertically on the silicon substrates with good alignment. MoS_2_ nanosheets grown on wafers using CVD are shown in Fig. 2b and d. The MoS_2_ nanosheets are distributed on the silicon substrate uniformly. The observable MoS_2_ film consists of vertically standing nanosheets. Abundant MoS_2_ nanosheet layers provide large quantities of edge sites, which are beneficial for gas sensing properties.

**Fig. 3:**
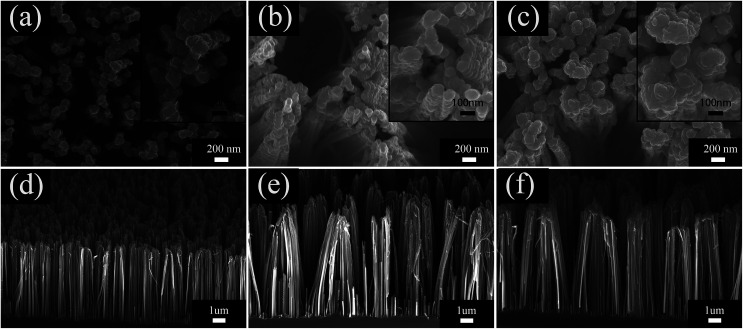
SEM images of top views of (a) MoS_2_/PSiNWs-1 min, (b) MoS_2_/PSiNWs-3 min and (c) MoS_2_/PSiNWs-5 min and side views of (d) MoS_2_/PSiNWs-1 min, (e) MoS_2_/PSiNWs-3 min and (f) MoS_2_/PSiNWs-5 min. Illustrations in (a–c) are high resolution SEM of top view.

Fig. 3 shows the thickness evolution and surface morphology of MoS_2_ nanosheets grown on PSiNWs using CVD. The deposition rate of MoS_2_ on the silicon wafers was ∼20 nm min^−1^. Fig. 3a and c show the top view and side view of edge-exposed MoS_2_ nanosheets on PSiNWs. The MoS_2_ nanosheets are grown densely on the top and lateral surfaces of the PSiNWs, which increases the reactive areas for gas molecules. The magnified view of the surface with obvious edges indicates the possibility of layer structure growth. An increase in the thicknesses of the MoS_2_ nanosheets seems to appear near the edges, where they begin to cluster (Fig. 3b and d). Fig. 3c and d show that isolated nanosheets are clustered together to form densely layered stacked structures. One can see that quantities of MoS_2_ nanosheets are interconnected with each other and generate many holes or channels for gas to quickly diffuse from the surface to the inside of the nanostructure. The distribution of MoS_2_ nanosheets on the lateral surface of PSiNWs was further examined using transmission electron microscopy (TEM).

**Fig. 4:**
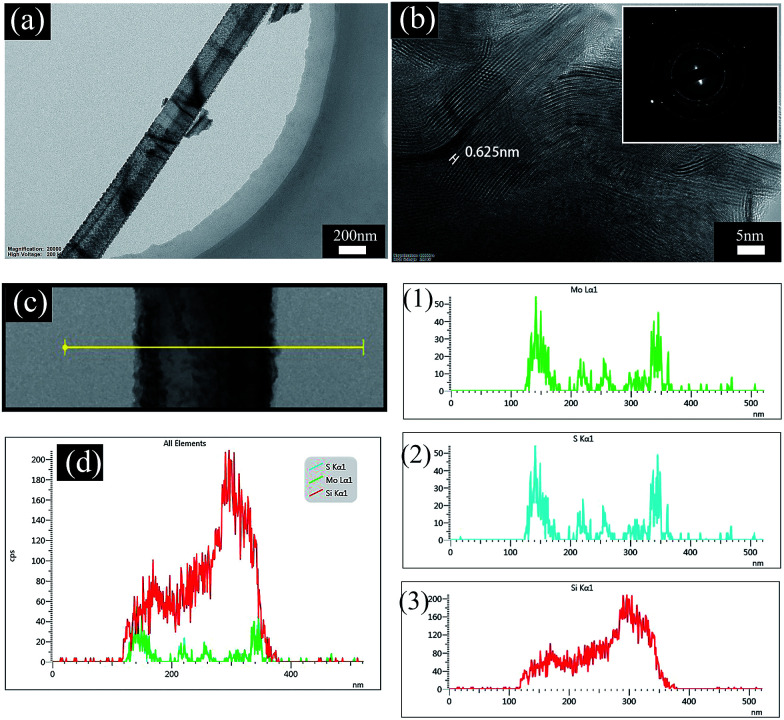
(a) The morphology of MoS_2_/PSiNW nanostructures. (b) The high-resolution TEM image and selective area electron diffraction (SAED) pattern. (c) The site of line scanning and (d) the elemental distribution of Si, Mo and S. Details for the elements Mo (1), S (2) and Si (3).

TEM was used to analyze the nanostructure and elemental distribution. Fig. 4a shows that the rod-like morphology still remains after forming MoS_2_ nanosheets on the PSiNW nanostructures. The MoS_2_ nanosheets are uniformly distributed on the surface of a single porous silicon nanowire. Fig. 4b clearly shows that each layer consists of an edge atomic structure with the S–Mo–S sequence, because the Mo atoms are heavier and appear brighter. The thickness of the Mo seed layer can change the horizontal and vertical alignment of MoS_2_.^22^ Many stripes are at the edges of MoS_2_ nanosheets, which reveal vertically aligned 2D MoS_2_ layers. Some grains are composed of a large number of self-assembled MoS_2_ layers with an interlayer spacing of 0.625 nm, which is consistent with the theoretical spacing for (002) planes of the hexagonal MoS_2_ nanostructures. A selective area electron diffraction (SAED) pattern (Fig. 4b inset) reveals that the MoS_2_/PSiNWs have a polycrystalline structure. The elemental distributions of Si, Mo and S are summarized in Fig. 4d. The summarized curve shows only the distribution of Mo and Si atoms, because distribution curves of Mo and S are coincident, which can be confirmed in Fig. 4 (1) and (2). Where a Mo atom is present, S atom will react with it to form MoS_2_. Si atoms are mainly distributed inside of the MoS_2_/PSiNW nanostructures, while Mo and S are distributed on the edges and surfaces.

**Fig. 5:**
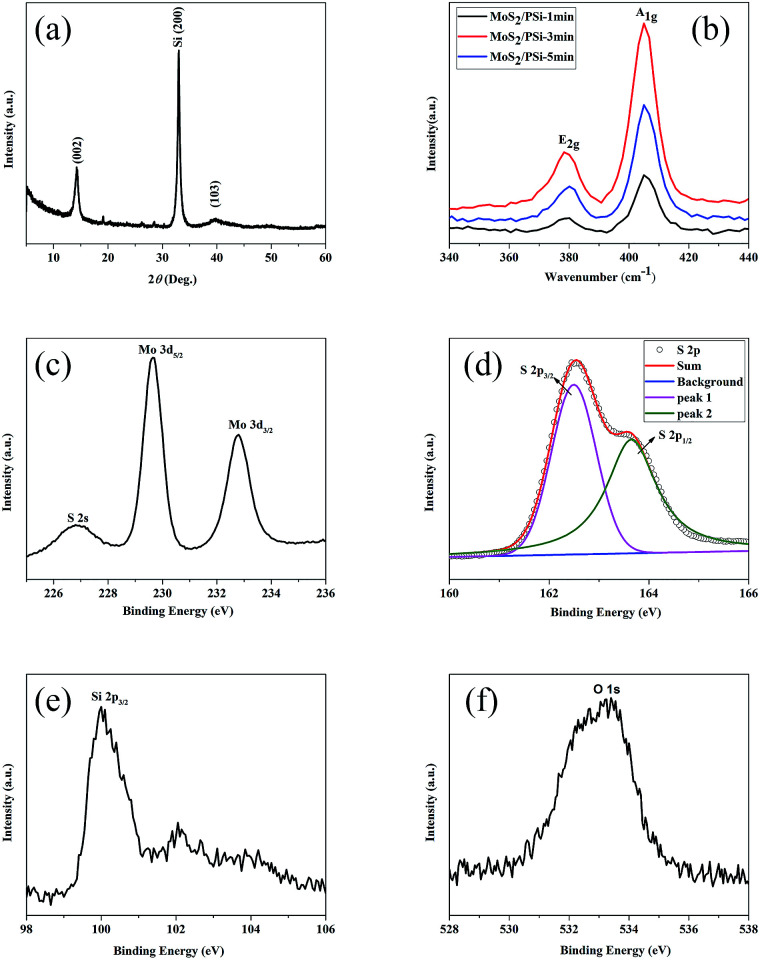
(a) X-ray diffraction (XRD) pattern of MoS_2_ nanosheets grown on PSiNWs. (b) Raman spectra of MoS_2_ on PSiNWs with two major peaks: E_2g_ and A_1g_ vibration modes. XPS spectra of (c) Mo 3d, (d) S 2p, (e) Si 2p and (f) O 1s in MoS_2_/PSiNW samples.

The structural details and phase purity of MoS_2_/PSiNW crystals were studied using XRD. All of the synthesized samples were measured with diffraction peaks in the range of 5–60°, as shown in Fig. 5a. The diffraction peaks of the MoS_2_ nanosheets are observed at 14.42° and 40°, which correspond to the (002) and (103) planes (JCPDS no. 37-1492). A strong peak at 33° that belongs to Si (200) was observed.

Raman spectra give many properties of the material, namely, structural features, orientation of the facets in the crystal, transition of the material, bonding details, thermal conductivity and compositional detail.^23^ As Fig. 5b shows, MoS_2_ has two major peaks: E_2g_ and A_1g_ vibrational modes of the Mo–S bonds near 380 cm^−1^ and 405 cm^−1^. E_2g_ represents the in-plane vibrational mode of the Mo and S atoms and A_1g_ represents the out-of-plane vibrational mode of S atoms.^24^ The peak position difference (*n*) between E_2g_ and A_1g_ is about 25 cm^−1^, which is consistent with those observed for bulk MoS_2_.^25^ The result is quite sensitive to the thickness of 2-D layered material systems, and that is consistent with what has been reported.^26,27^ Another feature observed in the Raman spectra is the intensity ratio of E_2g_/A_1g_, the value of which is approximately 0.6. The value indicates an obvious out-of-plane vibration (A_1g_) mode over in-plane vibration (E_2g_), reflecting the dominantly exposed MoS_2_ edge sites. The result proves it is typical vertical growth,^28,29^ which is consistent with the TEM result shown in Fig. 4b.

The surface composition and chemical states of the MoS_2_/PSiNW nanostructure were measured using X-ray photoelectron spectroscopy (XPS).^28^Fig. 5c exhibits the Mo 3d spectrum of the MoS_2_/PSiNW nanostructure. Two peaks at 229.65 and 232.75 eV are attributed to Mo 3d_5/2_ and Mo 3d_3/2_ of Mo^4+^.^30^ The other peak at 226.85 eV is in agreement with S^2−^ 2s.^31^ The S 2p spectrum in Fig. 5d shows two double peaks at 162.5 and 163.7 eV, which are ascribed to S 2p_3/2_ and S 2p_1/2_ of S^2−^, respectively.^32^ The binding energy is also consistent with previous reports (2p_3/2_: 162.4 eV and 2p_1/2_: 163.3–164.14 eV).^33–35^Fig. 5e shows that Si has two valence states. The binding energy of Si (2p_3/2_) observed at 99.95 eV indicates the existence of Si–Si. Fig. 5f shows the O 1s spectrum, indicating a peak at 533.05 eV corresponding to silicon oxide.

## 4. Gas studies

### 4.1. Gas-sensing properties

**Fig. 6:**
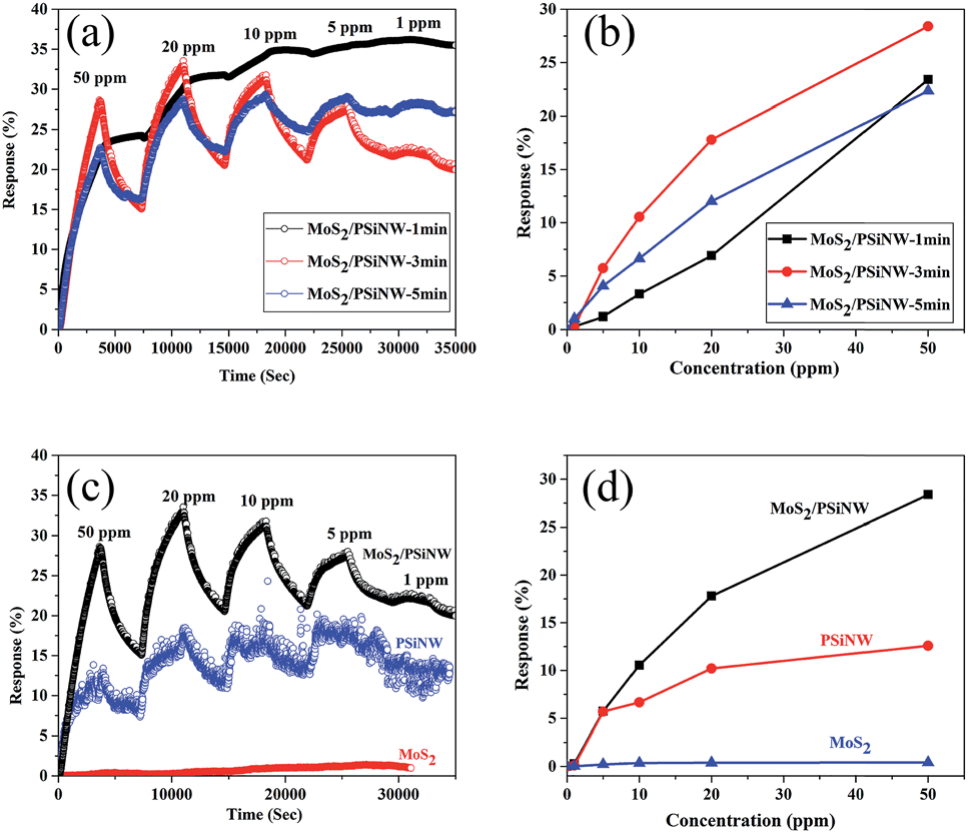
NO_2_ gas-sensing properties of MoS_2_/PSiNW nanostructures with different thicknesses of MoS_2_ nanosheets: (a) time-dependent gas-sensing behaviors toward different concentrations of NO_2_; (b) the response as a function of gas concentration. The study of gas properties with different substrates: (c) dynamic response in different NO_2_ concentrations; (d) response values of NO_2_ concentrations.

Fig. 6 shows the response and recovery curves of the MoS_2_/PSiNW heterojunctions with different thicknesses and substrates at room temperature. The sensitivity was measured upon sequential NO_2_ exposures in the range of 1–50 ppm. The time-dependent gas sensing behaviors toward different concentrations of NO_2_ are shown in Fig. 6a. MoS_2_/PSiNWs-3 min exhibited the most sensitivity to NO_2_, in comparison to MoS_2_/PSiNWs-1 min and MoS_2_/PSiNWs-5 min. The highest limit of detection was at 1 ppm. The recovery (60 min after exposure to NO_2_) could not be fully completed in cycles of 50–1 ppm. The phenomenon was also observed in a graphene-based sensor^36^ and in previous sensor reports.^37^Fig. 6b shows the sensing response as a function of gas concentration. The highest response values (MoS_2_/PSiNWs-3 min) of each cycle were 0.27, 5.72, 10.55, 17.8 and 28.4% with NO_2_ concentrations of 1, 5, 10, 20, 50 ppm. This study revealed the influence of the thickness of MoS_2_ on the gas sensing properties of MoS_2_/PSiNW heterojunctions. The different thicknesses of the MoS_2_ nanosheets on PSiNWs could change the energy levels of the conduction and valence bands, which has been shown in an earlier report.^38^Fig. 6c shows the dynamic response with different substrates, such as MoS_2_/PSiNWs, MoS_2_, and PSiNWs sensors. Among three sensors, MoS_2_/PSiNWs showed the highest response to NO_2_. Its response was nearly linear and increased by a factor of 2.3 in comparison to that of PSiNWs as shown in Fig. 6d. The formation of MoS_2_/PSiNW heterojunctions has improved the gas sensing properties greatly.

**Fig. 7:**
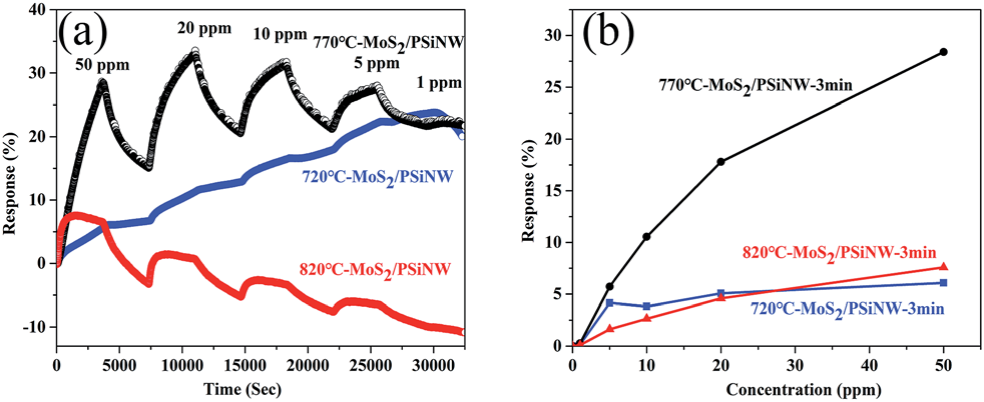
Gas sensing properties to NO_2_ of S-1, S-2 and S-3: (a) time-dependent gas-sensing behaviors toward different concentrations of NO_2_; (b) the response as a function of gas concentration.

Fig. 7a shows time-dependent gas-sensing behavior toward different concentrations of NO_2_. Compared with S-1 and S-3, S-2 exhibited a higher sensitivity to NO_2_. Moreover, S-1 and S-3 had no recovery at any cycle, while S-2 showed good recovery. The response value was further studied, and the results are shown in Fig. 7b. Compared with S-1 (6% to 50 ppm) and S-3 (7.7% to 50 ppm), S-2 (28.4% to 50 ppm) exhibited the highest response to NO_2_ and increased by a factor of 4. This phenomenon is attributed to the decreased defects and grain boundaries. With increasing temperature from 720 °C to 820 °C, the chemical reaction was accelerated and the adsorption and diffusion of sulfur molecules at the interface were strengthened, which increased crystallite size and decreased the grain boundaries (see ESI†). Moreover, the number of defects would be decreased. The crystallite size of MoS_2_ with the deposition temperature of 820 °C was too large, covering the surfaces of the MoS_2_/PSiNWs and perturbing interfacial gas molecule diffusion considerably at the molecule surface. The device with deposition temperature of 720 °C could not react completely, resulting in unstable structures. This could be explained by the rapid transfer charge in MoS_2_ and the high accumulation of electrons at the interface after increasing the annealing temperature.^39,40^ Therefore, the optimum deposition temperature was found to be 770 °C.

**Fig. 8:**
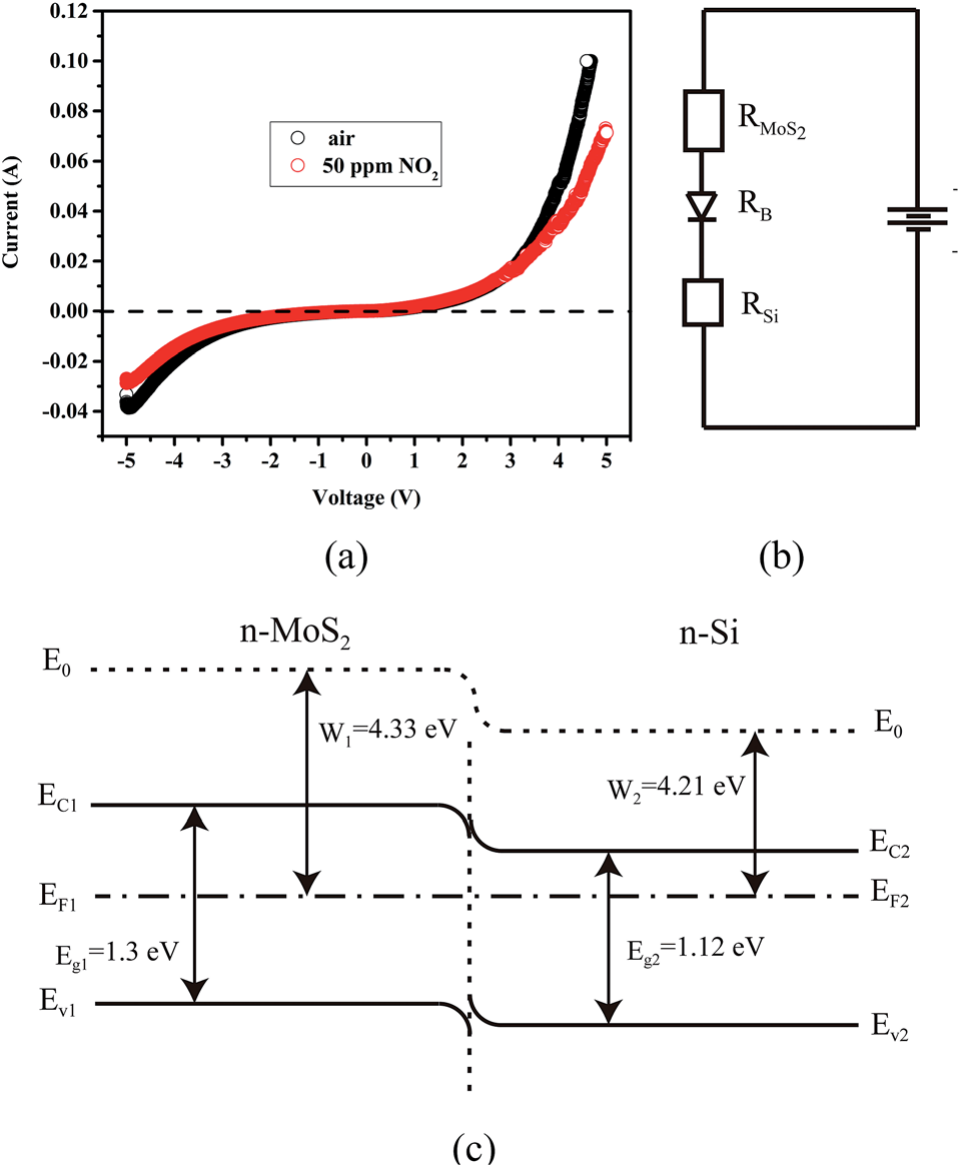
(a) *I*–*V* curves of MoS_2_/PSiNW heterojunctions in air and NO_2_. (b) Equivalent electrical resistance model of MoS_2_/PSiNW heterojunctions schematic illustration of using CVD to grow MoS_2_ nanosheets on PSiNWs. (c) Schematic illustration of the energy band of MoS_2_/PSiNW heterojunction structures.

Fig. 8a shows the current–voltage (*I*–*V*) curves of the MoS_2_/PSiNW heterojunction in dry air and NO_2_ gas. The measurements were performed 60 min later after the device was put under the above conditions. The *I*–*V* curves exhibited good rectification characteristics at room temperature. The rectification ratio (*I*_+_/*I*_−_) at the voltage of ±5 V for this device was about 2.6.

The MoS_2_–Ag contacts were ohmic in nature, therefore, the rectifying *I*–*V* characteristics were mainly attributed to the MoS_2_/PSiNW heterojunction. When the air conditions were changed to NO_2_, obvious changes in the *I*–*V* curves of the device were observed. As shown in the figure, the current decreased largely in both the forward and reserve directions. This demonstrates that the electrical properties of the junctions were dependent on NO_2_. Thus, this MoS_2_/PSiNW heterojunction device could function as a gas sensor. This is also confirmed in Table 1 by the comparison of the sensitivity to NO_2_ of other sensors based on different MoS_2_ nanostructures. The mechanism will be explained in detail later.

**Table 1:** Comparison of the sensitivity to NO_2_ gas of gas sensors based on different MoS_2_ nanostructures and similar materials

Sensing material	Fabrication method	*T* (°C)	NO_2_ (ppm)	Response (%)	Reference
MoS_2_/PSiNWs	Chemical etching + CVD	RT	50	28.4%	Present work
MoS_2_ film	CVD	RT	50	17.1%	41
rGO	Hydrolysis method	RT	50	10.8%	42
MoS_2_/carbon nanotube	CVD	RT	50	12.6%	43
MoS_2_/TiO_2_ nanotube	Anodization + hydrothermal method	150	50	14.2%	44
MoS_2_/Au	Drop-coating	60	50	2.2	45
rGO/ZnO	Spraying	RT	50	3.05	46
rGO/TiO_2_	Hydrothermal method	RT	50	15.9	47

### 4.2. Gas sensing mechanism

The above-mentioned results reveal that the MoS_2_/PSiNW heterojunctions exhibited good gas sensing properties to NO_2_ at room temperature. The high sensitivity is due to the layered nanostructures that induce more active sites for the absorption of NO_2_ and the modulation of changes to the band bending and depletion layer width at the interface. Moreover, MoS_2_ has a remarkable concentration of S vacancies (5%).^48^ When the sensor is exposed to nitrogen dioxide, NO_2_ molecules act as electron acceptors and form NO_2_^−^ (ads) through capturing free electrons from the conduction band of the MoS_2_ sheets. An electron depletion layer will be formed in the process, which leads to an increase in sensor resistance. When nitrogen gas contacts surface of the sensor, N_2_ molecules will react with nitrogen dioxide ions and release the trapped electrons back to the conduction band, decreasing the electron depletion layer width and increasing the resistance of the sensor. The reaction process can be shown as follows:

1 MoS_2_ + NO_2_ (gas) + e^−^ = MoS_2_ + NO_2_^−^ (ads)

2 2NO_2_^−^ (ads) + N_2_ → 4NO + 2e^−^

The work function and the band gap of n-Si are 4.21 and 1.12 eV, respectively, while the work function and the band gap of MoS_2_ are 1.3 and 4.33 eV, respectively. The Fermi level of n-Si is higher than that of MoS_2_ and thus the electrons transfer through the interface from n-Si to MoS_2_ until their Fermi levels equalize, as shown in Fig. 8c. When the device is exposed to NO_2_, larger amounts of NO_2_ molecules are absorbed on the surface of the MoS_2_ film. Subsequently, some NO_2_ molecules can be injected into the whole layers of film through the grain boundaries, and even reach the interface area of the junction. NO_2_ captures free electrons from the conduction band of MoS_2_ sheets, leading to a reduction in the charge carriers in it,^49,50^ and increasing the resistance of MoS_2_. A depletion layer can be formed at the interface of MoS_2_/PSiNWs. Moreover, the Fermi levels of the MoS_2_ films shift toward the valence band as has been previously reported,^51^ and the energy barrier increases at the interface of MoS_2_/PSiNWs. The sensing mechanism can be explained by the equivalent electrical circuit, as shown in Fig. 8b. The resistance of the MoS_2_/PSiNW heterojunctions is composed of the resistance of the MoS_2_ (*R*_MoS_2__), the barrier (*R*_B_) and the PSiNWs (*R*_Si_).^52^*R*_Si_ does not show any obvious change after exposure to NO_2_ due to the heavy doping of Si (0.01–0.05 Ω × cm). *R*_MoS_2__ and *R*_B_ mainly determine the resistance variation of the MoS_2_/PSiNW heterojunction. Fig. 8c shows an obvious potential barrier in the device. The properties of this barrier can greatly affect the resistance of the device as a result of its exponential relationship.^53^ After adsorption, the charge transfer will lead to a low density of electrons on the MoS_2_ side in the heterojunction, which increases the barrier of the heterojunction (*R*_B_). This is confirmed by the *I*–*V* curve in Fig. 8a. It has been reported that the variation of barrier height and width due to gas adsorption can significantly change the resistance.^54,55^ Therefore, MoS_2_/PSiNWs exhibit superior gas sensing properties compared to MoS_2_ and PSiNW sensors (Fig. 6c).

## 5. Conclusion

We demonstrate a new and simple fabrication method of MoS_2_/PSiNW heterojunctions. In this approach, PSiNWs were obtained *via* Ag-assisted chemical etching, and then MoS_2_ nanosheets were synthesized using sulfurization of direct-current (DC)-magnetic-sputtering Mo films on PSiNWs. The MoS_2_/PSiNW heterojunctions exhibit superior gas sensing properties with a high response enhancement factor of ∼2.3 at room temperature, in comparison with MoS_2_ and PSiNWs. The MoS_2_/PSiNWs-3 min with MoS_2_ thickness of ∼60 nm showed a maximum response of 28.4% to 50 ppm NO_2_ and a highest limit of detection at 1 ppm. Additionally, MoS_2_/PSiNWs fabricated using different deposition temperatures in the chemical vapor deposition (CVD) process were also measured and the results show that the optimum deposition temperature was 770 °C. The enhancement in gas sensing performances is attributed to the predominant geometrical morphology and effect of the depletion layer width at the interface. Moreover, a remarkable concentration of S vacancies in MoS_2_ acted as independent active sites, magnifying the gas sensing properties of PSiNWs, by improving the interaction of molecules with defects. Therefore, MoS_2_/PSiNW heterojunctions could stimulate greater innovation for future sensor technologies.

## Conflicts of interest

There are no conflicts to declare.

## Supplementary Material

RA-008-C7RA13484C-s001
