# Supplementary material for: Highly enhanced response of MoS2/porous silicon nanowire heterojunctions to NO2 at room temperature
Source: RSC Adv. 2018 Mar 21;8(20):11070–7. doi: 10.1039/c7ra13484c (PMC9078941; doi:10.1039/c7ra13484c)
Supplement: RA-008-C7RA13484C-s001 [file RA-008-C7RA13484C-s001.pdf]

Electronic Supplementary Material (ESI) for RSC Advances.
This journal is © The Royal Society of Chemistry 2018

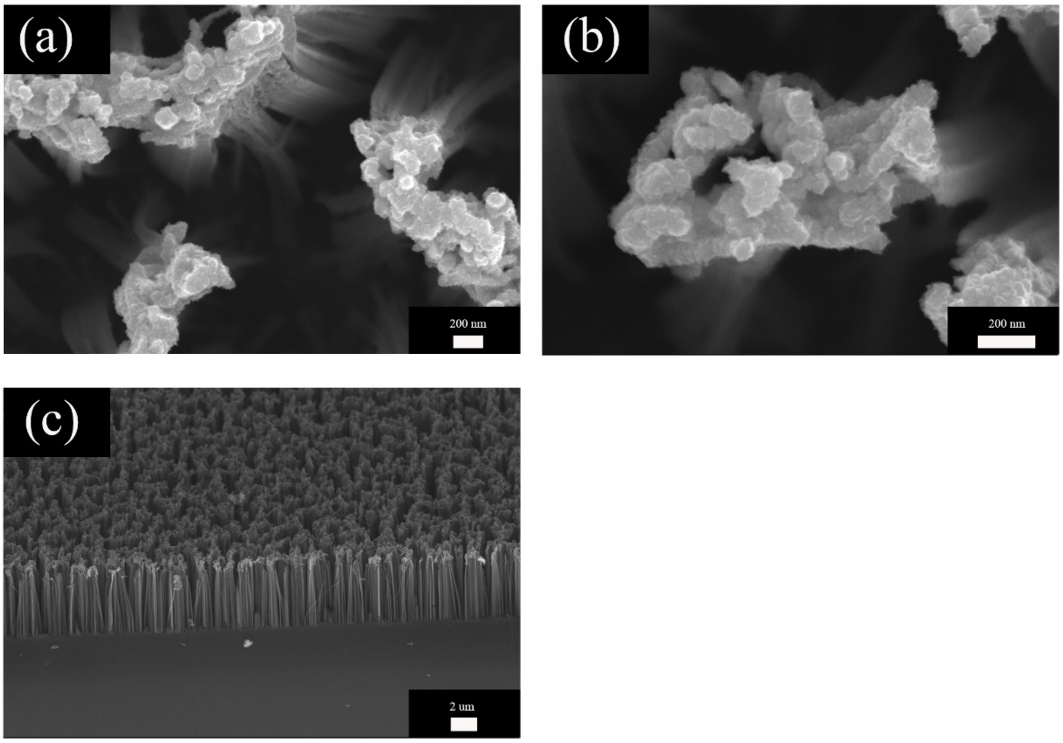

Figure 1. SEM images of MoS2/PSiNWs-3 min with deposition temperature of 720 ℃. Side view (a) and top view (b), (c).

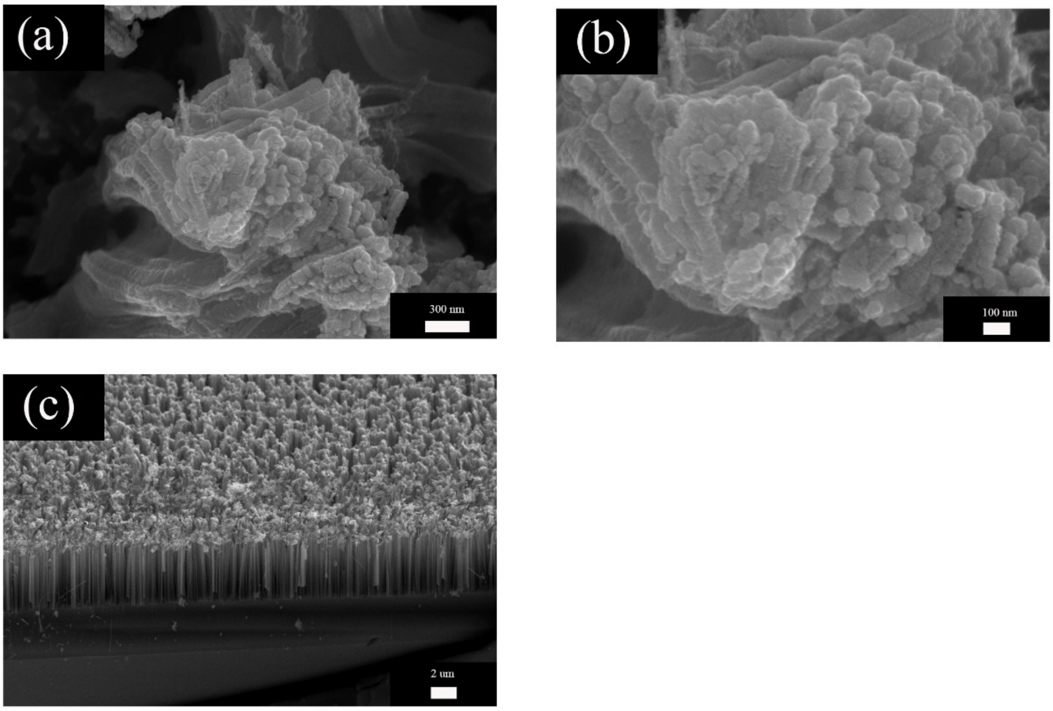

Figure 2. SEM images of MoS2/PSiNWs-3 min with deposition temperature of 820 ℃. Side view (a) and top view (b), (c).
